# What morphological MRI features enable differentiation of low-grade from high-grade soft tissue sarcoma?

**DOI:** 10.1259/bjro.20210081

**Published:** 2022-06-22

**Authors:** Sana Boudabbous, Marion Hamard, Essia Saiji, Karel Gorican, Pierre-Alexandre Poletti, Minerva Becker, Angeliki Neroladaki

**Affiliations:** ^1^ Division of Clinical Pathology, Diagnostic Department, Geneva University Hospitals, University of Geneva, Geneva, Switzerland; ^2^ Division of Radiology, Diagnostic Department, Geneva University Hospitals, University of Geneva, Geneva, Switzerland

## Abstract

**Objective:**

To assess the diagnostic performance of morphological MRI features separately and in combination for distinguishing low- from high-grade soft tissue sarcoma (STS).

**Methods and materials:**

We retrospectively analysed pre-treatment MRI examinations with T1, T2 with and without fat suppression (FS) and contrast-enhanced T1 obtained in 64 patients with STS categorized histologically as low (*n* = 21) versus high grade (*n* = 43). Two musculoskeletal radiologists blinded to histology evaluated MRI features. Diagnostic performance was calculated for each reader and for MRI features showing significant association with histology (*p* < 0.05). Logistic regression analysis was performed to develop a diagnostic model to identify high-grade STS.

**Results:**

Among all evaluated MRI features, only six features had adequate interobserver reproducibility (kappa>0.5). Multivariate logistic regression analysis revealed a significant association with tumour grade for lesion heterogeneity on FS images, intratumoural enhancement≥51% of tumour volume and peritumoural enhancement for both readers (*p* < 0.05). For both readers, the presence of each of the three features yielded odds ratios for high grade versus low grade from 4.4 to 9.1 (*p* < 0.05). The sum of the positive features for each reader independent of reader expertise yielded areas under the curve (AUCs) > 0.8. The presence of ≥2 positive features indicated a high risk for high-grade sarcoma, whereas ≤1 positive feature indicated a low-to-moderate risk

**Conclusion:**

A diagnostic MRI score based on tumour heterogeneity, intratumoural and peritumoural enhancement enables identification of lesions that are likely to be high-grade as opposed to low-grade STS.

**Advances in knowledge:**

Tumour heterogeneity in Fat Suppression sequence, intratumoural and peritumoural enhancement is identified as signs of high-grade sarcoma.

## Introduction

Soft tissue sarcomas (STS) are a heterogeneous group of malignancies with more than 50 histologic subtypes and a high mortality rate^1^ Although relatively rare as representing only 1% of adult malignancies,^2^ their diagnosis remains challenging in terms of detection, differentiation from benign lesions and pre-treatment classification with MRI into high- versus low-grade tumours, the histopathologic grade being one of the most important prognostic factors.^3,4^ The differentiation between low- versus high-grade STS also affects initial treatment as high-grade lesions require neo-adjuvant chemotherapy or radiotherapy before surgical resection.

Although the accurate initial diagnosis – primordial for treatment – is invariably based on histopathologic analysis, imaging is essential to orient towards an adequate therapeutic choice and to guide biopsy.

MRI is currently the imaging modality of choice for precise tumour localisation, assessment of tumour relationship to major anatomic landmarks and for the evaluation of tumour architecture and vascularisation. To the best of our knowledge, only few studies have so far addressed the diagnostic value of morphological MRI to distinguish low- from high-grade STS. These studies have focussed on peri-tumoural enhancement and signal, both features having been shown to be related to tumour grade,^5–7^ as well as on tumour necrosis and intratumoural heterogeneity.^8^ Other studies have evaluated the added value of advanced functional MRI techniques including perfusion and diffusion-weighted imaging.^9,10^ However, functional MRI techniques are not always routinely performed in the initial lesion work-up, and morphological MRI sequences still remain the “working horse” in many institutions worldwide.

The aim of our study was to evaluate the diagnostic performance of reproducible morphological MRI features that separately or in combination can be used to predict the correct tumour grade.

## Methods and materials

### Study design

This retrospective study was performed after local Institutional Review Board approval and in accordance with the guidelines of the Helsinki declaration. Informed consent was waived.

**Figure 1.**
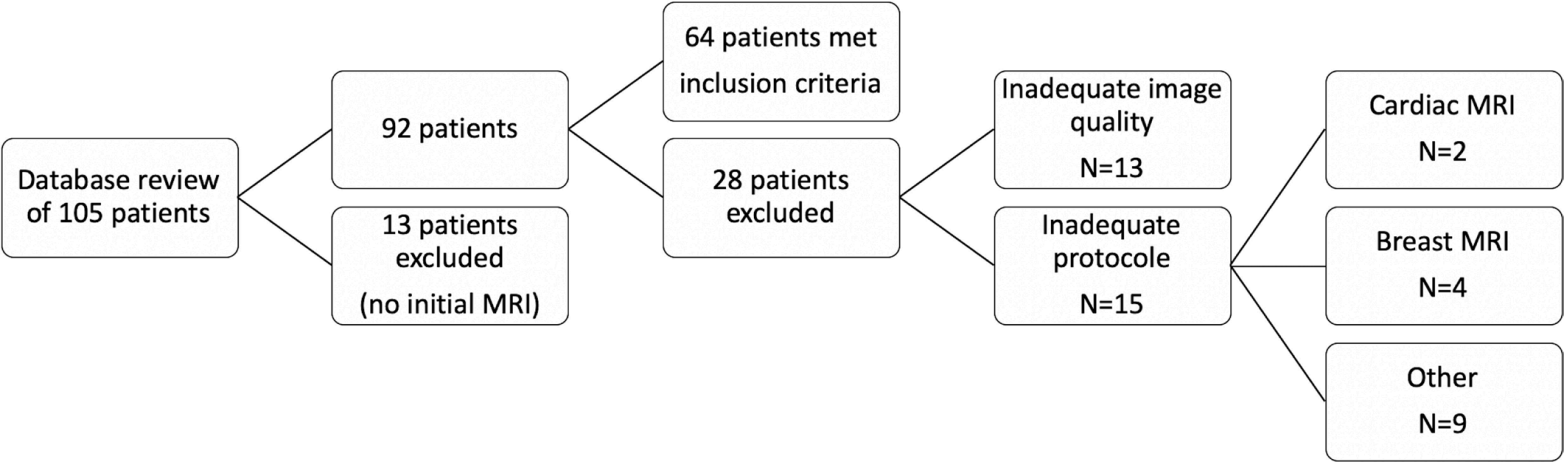
Diagram of inclusion and exclusion criteria of the patients

Study population:

We reviewed our institutional sarcoma board records to identify all patients with histologically confirmed STS and imaged with MRI between March 2010 and August 2018. Our institution is a tertiary referral centre and MRI examinations of patients presented at the interdisciplinary sarcoma board are, in part, done in outside institutions and, therefore, on different MRI machines. From a total of 105 patients with STS, 64 were eligible for our study (Figure 1).

Inclusion criteria were as follows: a) STS of the trunk or limbs proven by histopathological analysis of specimens from surgical ablation/biopsy or percutaneous biopsy; b) pre-treatment/pre-biopsy MRI with the following sequences: T1, T2 with fat suppression (FS) technique (T2 with FS, PD with FS, short tau inversion recovery sequence) and contrast-enhanced T1 images. Exclusion criteria were inadequate MRI protocol (lacking≥one of the above-mentioned sequences), non-diagnostic image quality and no precise histopathologic diagnosis. Therefore, we excluded 41 patients, due to the following reasons: absence of initial MRI (*n* = 13), inadequate image quality on≥one sequence (*n* = 13), MRI protocol not meeting the inclusion criteria in cardiac (*n* = 2), breast (*n* = 4), peritoneal and intra-abdominal extraperitoneal sarcomas (*n* = 9).

Patient demographics including age, gender, final histopathologic diagnosis, sarcoma grading and tumour location were recorded. According to the French Federation of Cancer Centres grading system, tumours were categorised as low grade (Grade 1) and high grade (Grade 2–3) based on histologic specimens from percutaneous biopsy (*n* = 24), surgical biopsy (*n* = 9) or surgical resection (*n* = 31).^11^

### Image analysis

**Figure 2.**
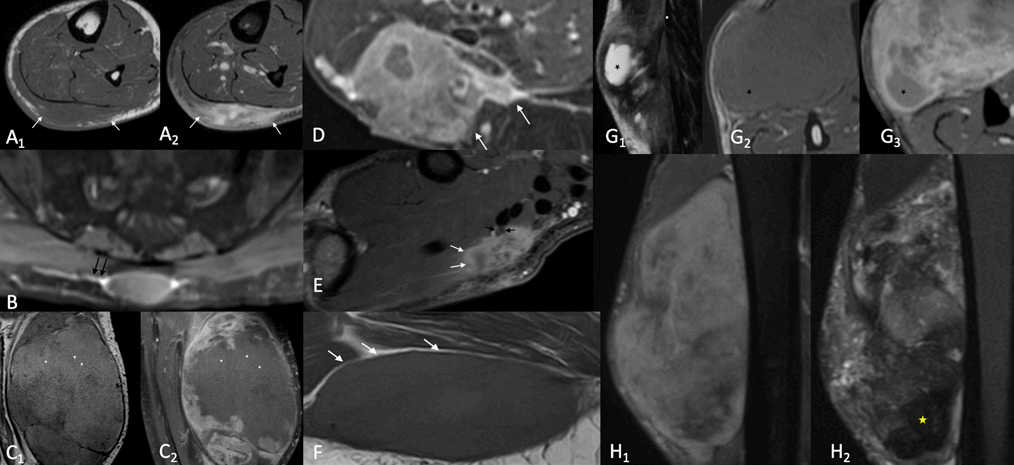
Illustration of MRI features analysed by the two readers: A) Important tumoural enhancement (≥51% of tumour volume) on contrast enhanced FS T1 (A2) and diffuse growth pattern on both T1 (A1) and contrast enhanced FS T1 (A2) (arrows) B) Fascia tail sign (black arrows) on contrast enhanced FS T1. C) Tumoural haemorrhage: on T1 weighted images (C1) areas of hyperintense signal (asterisks) presenting no enhancement (asterisks) on contrast enhanced FS T1 (C2) D) Peritumoural enhancement (white arrows) on contrast enhanced FS T1 E) Diffuse growth pattern (arrows) and encasement of the thenar branch of the median nerve (black arrows) F) Peritumoural capsule fat sign (white arrows) G) Tumoural necrosis (asterisk): hyperintense signal on T2 (G1) hypointense signal on T1 (G2) and no enhancement on contrast enhanced FS T1 (G3). H) Highly heterogeneous lesion (more than 2/3 of the volume of the mass) on STIR (H1) presenting zones of hypointense signal on T2 (H2)

Two board-certified radiologists with nine and two years of experience in musculoskeletal radiology after board certification reviewed the MR images blinded to sarcoma subtype and histologic grade. They recorded lesion size (largest diameter in cm), precise location and whether the lesion was deep or superficial to the muscle fascia. Lesions were considered as superficial if located in the subcutaneous layer, whereas lesions with intra/intermuscular location and lesions with both subcutaneous and profound location were considered as deep. Tumours were classified on each sequence separately as homogeneous if <1/3 of the lesion was heterogeneous and as heterogeneous if >2/3 of the lesion was heterogeneous. Tumour margins were categorised as ill-defined (>10–25% of tumour margins blurred), and well-defined (>90% of margins clearly delimitated). Tumoural enhancement was estimated as percentages of overall tumour volume. The peripheral growth pattern was categorised as focal (well delineated margins without surrounding invasion), and diffuse (tumour invasion of surrounding structures either partly or along the entire tumour circumference). The presence/absence of the following features was equally recorded: neurovascular bundle encasement, haemorrhage, necrosis, internal low signal elements on *T_2_*-weighted sequences, fascia tail sign, peritumoural capsule fat sign and lymphadenopathy (Figure 2).

The following criteria were considered as indicating high-grade malignancy: largest lesion diameter ≥5 cm, deep location, lesion heterogeneity in ≥2/3 of total tumour volume, intratumoural enhancement in ≥51% of tumour volume, presence of intratumoural haemorrhage, presence of necrosis, areas of low signal on *T_2_*-weighted images, diffuse growth pattern, fascia tail sign, lymphadenopathy, neurovascular bundle encasement, absent peritumoural capsule fat sign, blurred tumour margins and peritumoural enhancement^6,12^ Table 1.

**Table 1.** Multivariate logistic regression models for high-grade sarcoma, for each reader, with areas under the ROC curve

	Reader 1		Reader 2	
	Odds ratio	P	Odds ratio	P
**Lesion heterogeneity in ≥2/3 of total tumour volume on FS images**	5.2 (1.1–23.9)	0.035	5.1 (1.1–24.2)	0.040
**Tumour enhancement (>51%) on contrast enhanced T1**	4.4 (1.0–18.8)	0.044	6.8 (1.6–28.9)	0.010
**Presence of peritumoural enhancement on contrast enhanced T1**	4.9 (1.2–19.8)	0.025	9.1 (2.1–38.7)	0.003
	AUC		AUC	
Logit score	0.826		0.849	
Sum of positive items	0.812		0.849	

### Statistical analysis

Sensitivity and specificity for differentiating low- from high-grade tumours were calculated for each reader and each feature, respectively. The association between each MRI feature and STS grade was evaluated using a chi-square test.

κ statistics for all morphological MRI features were used to assess inter-rater reproducibility (reader 1 versus reader 2). MRI features sufficiently reproducible (kappa>0.5) were used to develop a diagnostic model using multiple regression logistic analysis separately for the two readers. An analyst-controlled procedure was used starting with the strongest univariate predictor. Areas under receiver operating characteristic curves (AUC) were obtained for the corresponding logistic equation and for the sums of positive items to assess the discrimination of each model. The summary scores across tumour stage were cross-tabulated, separately for the two readers. The two scores were dichotomised and their sensitivity specificity, and positive and negative predictive values were calculated. Inter-rater intraclass correlation coefficients (ICC) for the logistic scores and for the sum of positive signs were obtained. The analysis was performed with IBM SPSS version 25.

## Results

Patient data and tumour characteristics are shown in Table 2 . Descriptive statistics for MRI features for each reader is shown in in Supplementary Material 1. Table 3 illustrates the diagnostic performance for each MRI feature and for each reader and the interobserver agreement.

**Table 2.** Patient and tumour characteristics

Total No of patients	64	
**Female (No) (%**)	20 (31%)	
**Male (No) (%**)	44 (69%)	
**Mean age (years**)	64	
**Sarcoma grade**		
Low grade (=1) (No) (%)	21 (33%)	
High grade (≥2) (No) (%)	43 (67%)	
**Histologic subtypes (No) (%**)	**High grade** (**No) (%**)	**Low grade** (**No) (%**)
Liposarcoma (24) (38%)	8 (33%)	16 (67%)
Malignant peripheral nerve sheath tumour (2) (3%)	2 (100%)	0
Synovial sarcoma (5) (8%)	4 (80%)	1 (20%)
Fibromyxoid sarcoma (4) (6.5%)	3 (75%)	1 (25%)
Leiomyosarcoma (6) (9%)	4 (80%)	2 (20%)
Dermatofibrosarcoma (1) (1.5%	0	1 (100%)
Fusocellular sarcoma (4) (6.5%)	4 (100%)	0
Pleomorphic sarcoma (10) (16%)	10 (100%)	0
Pleomorphic sarcoma-myxoid components (1) (1.5%)	1 (100%)	0
Pleomorphic sarcoma with fusocellular components (4)	4 (100%)	0
Rhabdomyosarcoma (1)	1 (100%)	0

**Table 3.** Associations of MRI signs with high-grade sarcoma for the two readers

		Reader 1		Reader 2		
	Value	% High	p	% High	p	κ
**Deep layer location**	0 1	73.7 64.4	0.47	73.7 64.4	0.47	1
**Tumour size (>5 cm**)	0 1	76.2 62.8	0.28	77.3 61.9	0.21	0.90
**Lesion heterogeneity in ≥2/3 of total tumour volume on T1**	0 1	64.2 81.8	0.26	68.8 62.5	0.64	0.58
**Lesion heterogeneity in ≥2/3 of total tumour volume on FS images**	0 1	56.8 84.6	0.019^*^	58.8 76.7	0.13	0.62
**Lesion heterogeneity in ≥2/3 of total tumour volume on T1 FS images**	0 1	54.5 79.3	0.040^*^	56.8 80.8	0.047^*^	0.35
**Tumour enhancement (>51%) on contrast enhanced T1 images**	0 1	44.0 81.1	0.002^*^	44.4 82.9	0.002^*^	0.67
**Tumour haemorrhage**	0 1	57.5 83.3	0.033^*^	58.0 100	0.003^*^	0.20
**Tumour necrosis**	0 1	40.0 69.5	0.18	55.6 69.1	0.42	0.21
**Areas of low signal on T2**	0 1	44.4 83.8	0.001^*^	38.9 78.3	0.003^*^	0.30
**Present fascia tail sign**	0 1	37.0 89.2	<0.001^*^	55.9 80.0	0.04^*^	0.23
**Absent peritumoural capsule fat sign**	0 1	72.2 65.2	0.59	70.3 63.0	0.54	0
**Diffuse growth pattern**	0 1	65.1 71.4	0.61	68.8 65.6	0.79	0.41
**Blurred tumour margins**	0 1	47.6 76.7	0.020^*^	55.6 71.7	0.22	0.38
**Presence of peritumoural enhancement on contrast enhanced T1**	0 1	45.0 82.5	0.003^*^	42.1 82.4	0.002^*^	0.66
**Presence of neurovascular invasion**	0 1	69.4 60.0	0.50	65.3 73.3	0.56	0.48
**Lymphadenopathy**	0 1	63.8 100	0.072	64.8 80.0	0.35	0.01

**Figure 3.**
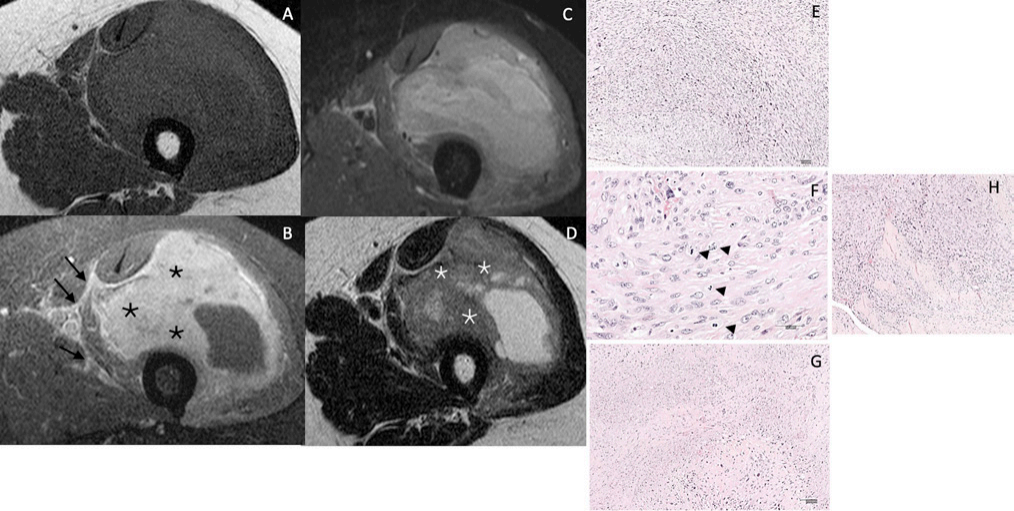
65 year old male patient with important swelling of the left thigh. T1 (A), contrast enhanced FS T1 (B), STIR (C), T2 weighted sequence (D). Major intratumoral enhancement (≥ 51% of tumour volume (black asterisks), peritumoural enhancement (arrows) and heterogenous signal on STIR, indicating high grade tumour. Internal areas of low T2 signal (white asterisks). Surgical biopsy revealed a high-grade leiomyosarcoma grade III by FNCLCC Intersecting fascicles of pleomorphc spindle cells (E-Hematoxylin and eosin, H&E, original magnification × 100), with high rate of mitotic figures (arrows) (F- H&E, original magnification × 400), and large areas of necrosis (G- H&E, original magnification × 100). Infiltrating aponevrotic tissu in the periphery of the tumor (H- H&E, original magnification × 100)

**Figure 4.**
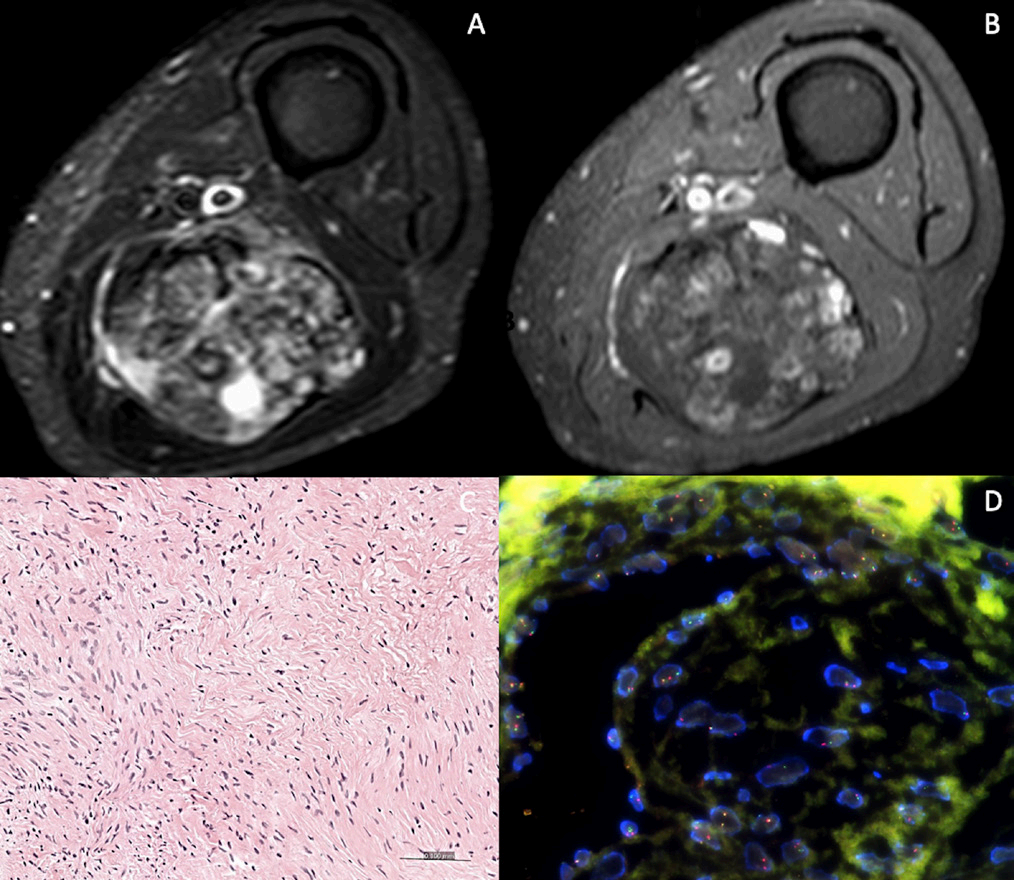
86 year old female patient with a palpable mass of the posterior lower left thigh discovered on MRI. STIR (A) and contrast enhanced FS T1 (B). Highly heterogenous mass on STIR, but tumoural enhancement <51% of the total volume (arrows) and no peritumoral enhancement. Surgical biopsy revealed a low grade fibromyxoid sarcoma (C- Hematoxylin and eosin, H&E, original magnification × 200) Scattered hyperchromatic cells admixed with heavily collagenized areas. No mitoses nor necrosis were seen. (D- By FISH- Fluorence in situ hybridization) FUS Dual Color Break Apart Probe detected a translocation involving the FUS gene.

Among the different MRI features evaluated, six items had acceptable reproducibility (kappa>0.5); size, deep layer localisation, lesion heterogeneity in ≥2/3 of total tumour volume on T1 and on FS images, intratumoural enhancement ≥51% of the tumour volume and peritumoural enhancement on contrast enhanced T1 images. Three signs showed an association with tumour grade for at least one reader; lesion heterogeneity in ≥2/3 of total tumour volume on FS images, intratumoural enhancement ≥51% of the tumour volume and peritumoural enhancement on contrast enhanced T1. The presence of two or three signs indicated a high risk for high-grade sarcoma (Figure 3), whereas zero or one sign indicated low-to-moderate risk for high-grade sarcoma (Figure 4).

These three signs were also associated with high-grade tumour in the multivariate regression logistic model with AUCs all above 0.8 Table 1 with good gradient of risks Table 4 and acceptable sensitivity and specificity for both models Table 5. The inter-rater ICC was 0.757 for the logistic scores and 0.725 for the sums of the three items Table 6.

**Table 4.** Distributions of summary scores, and proportions of patients with high-grade sarcoma

	Reader 1		Reader 2	
Score	N (%)	% high grade	N (%)	% high grade
0	7 (12.1)	14.3%	4 (6.7)	0.0%
1	13 (22.4)	46.2%	19 (31.7)	42.1%
2	25 (43.1)	84.0%	25 (41.7)	88.0%
3	13 (22.4)	92.3%	12 (20.0)	91.7%

**Table 5.** Test characteristics for the detection of high-grade sarcoma, for 2–3 positive signs versus 0–1 positive signs, for two readers

	Sensitivity	Specificity	Positive predictive value	Negative predictive value
Reader 1, ≥ 2	82.5%	72.2%	86.8%	65.0%
Reader 2, ≥ 2	80.5%	78.9%	89.2%	65.2%

**Table 6.** Summarizes MRI features correlated with risk of high -grade STS

MRI features			Risk of high-grade STS	
Lesion heterogeneity in ≥2/3 of total tumour volume on FS images	Tumour enhancement (>51%) on contraste-enhanced T1	Presence of peritumoural enhancement on contrast-enhanced T1	**High** two or three signs	**Low/moderate** 0 or one sign
				

## Discussion

In this retrospective study, we analysed the predictive morphologic MRI features allowing to differentiate low- from high-grade STS independently of the expertise of the interpreting radiologist.

Determining sarcoma grade is of high importance for treatment choice and for overall survival, sarcoma grade being considered the most important prognostic factor.^3^ For high-grade sarcomas, neo-adjuvant treatment, chemotherapy or radiotherapy are essential. Sarcoma grade is determined histopathologically on specimen obtained from surgical resection, biopsy or percutaneous biopsy. However, indicating the presumed tumour grade by imaging prior to biopsy is essential to decide, what is the most appropriate biopsy site and in order to complement histologic results, which can sometimes be non-conclusive. The histologic features determining sarcoma grade are tumour differentiation, necrosis and number of mitoses.^13^

MRI is the imaging modality of choice to determine intralesional characteristics (cystic versus solid, fatty, myxoid or vascular nature). However, predicting the exact histologic type of a malignant lesion has been shown to have a low accuracy^4,14^ and few studies have attempted to analyse morphological MRI features to predict tumour grade.^4,6–8,15^

Our study suggests a simple diagnostic tool including tumoural heterogeneity, intratumoural and peritumoural enhancement to identify high-grade sarcoma; features also described in other studies.^4,6–8,15^

In our series, intratumoural enhancement ≥51% of tumour volume was significantly correlated with high-grade tumours. Chhabra et al reported significantly more central enhancement with Grade III tumours and Zhao et al noticed a tendency towards more important tumour enhancement with high-grade sarcomas.^6,7^

Similar to our results, Zhao et al^6^ reported that peritumoural enhancement can be used to diagnose high-grade sarcomas, peripheral neovascularity indicating aggressive growth.^6^ Crombé et al also reported peritumoural enhancement as an independent predictor of tumour grade, associated with Grade III tumours.^8^ These findings confirm the necessity to obtain contrast-enhanced T1, as part of any protocol for soft tissue mass characterisation.

Tumour heterogeneity on FS sequences in >2/3 of the tumour mass was also included in the diagnostic model, and as intratumoural haemorrhage were significantly correlated with high-grade tumours. This could be explained by the fact that high tumour heterogeneity, which is a characteristic of aggressive tumours, relates to a mix of viable hypercellular zones, necrotic, haemorrhagic and fibrotic tissue; the tumoural heterogeneity has been investigated by more advanced techniques in the literature.^16,17^

Internal low signal areas on T2 indicate fibro-collagenous content, zones of hypocellularity, hemosiderin or calcified contents^18,19^ ; according to the literature, these can be detected both in benign and malignant tumours (fibrosarcoma, malignant fibrous histiocytoma, synovial sarcoma^19^ and typically in liposarcoma).^20–22^ In our study with a large number of liposarcomas, this feature was statistically significant for both readers and had a good correlation with high-grade tumours indicating again important heterogeneity, as a feature of aggressive tumours.

The fascia tail sign has been described not only as a sign for histologically aggressive tumours *e.g.,* myxofibrosarcomas^23^ but also as a sign of nonaggressive lesions, *e.g.,* peripheral nerve sheath tumours^24^ and desmoids.^25,26^ When related to myxofibrosarcoma, the fascia tail sign was moderately specific and sensitive for diagnosis.^23^ In our study, for both readers, this sign was significantly correlated with high-grade sarcomas (*p* = 0.001 and *p* = 0.04), probably indicating the tendency of the aggressive lesions to spread to the surrounding tissues.

However, only for reader 1, poorly defined tumour margins identified on MRI with ≥10–25% blurred margin predicted high-grade histologically. Assessing tumoural margins is challenging; it depends on tumour location and on the ability to differentiate tumour from the surrounding normal tissues. Reader experience may explain the limited sensitivity of reader two for high-grade STS applying this criterion. Fenebro et al evaluated the focal versus infiltrative tumour growth pattern and concluded that infiltrative growth >25% of tumour circumference was related to metastatic disease and local recurrence.^5^

As opposed to Liu et al, in our study, the peritumoural capsule sign was not a feature indicating low-grade tumours. Furthermore, vascular invasion and abnormal regional lymph nodes did not show significant correlation to predict high-grade tumours, however they were highly specific.

As opposed to earlier studies,^7,12,15^ we observed that tumour size (largest diameter≥5 cm) and deep location were not strongly related to high grade. Our data are in accordance with the recent literature, as the cut-off value of 5 cm has been shown to have a poor specificity and poor positive predictive value.^27^

In contrast to other studies,^8,15^ necrosis was not associated with high-grade tumour either for reader 1 or 2. This fact might be due to the difficulty of discriminating necrosis from cystic degeneration or myxoid component. Moreover, it might be related to our study population, one-third of lesions being liposarcomas (33%) including low-grade tumours that can present various degrees of fat necrosis and myxoid change.^22,28^

Limitations of the current study include retrospective design with no standardised MRI protocols resulting to an heterogenous dataset as MRI exams were obtained in different centres. The diagnostic score was constructed on a small sample size, and the risk exists that the model is overfitted. It would be useful to verify the properties of this score in an independent sample. Furthermore, the histologic grade for vast majority of cases was obtained after surgical resection/biopsy but in 24 cases histology was based on percutaneous biopsy, which may be related to underestimating sarcoma grade due to insufficient sampling^29,30^ ; nevertheless, only three of these cases were low-grade sarcomas, the other being high-grade tumours (most of them with histologic confirmation on the resection specimen after radio or chemotherapy), the risk of underestimating sarcoma grade being, therefore, low. Almost one-third of the cases in our study were liposarcomas, which display specific imaging patterns. Our study had more high-grade sarcomas than low-grade sarcomas, the prevalence of high-grade tumours possibly influencing the power of statistical results.

There was no standardisation or consensus between the readers, with different degrees of experience, therefore, possibly explaining the fact that some of the features were not sufficiently reliable (kappa<0.5) so as to develop the diagnostic score.

Finally, our study was focused strictly on the analysis of morphological features on basic MR sequences. Evaluation of functional MR techniques as diffusion-weighted imaging, dynamic contrast-enhanced imaging and correlation with FDG PET CT could strengthen the reliability on distinguishing low- from high-grade sarcomas. Diffusion-weighted imaging, a non-contrast functional method can be useful in tumour characterisation; lower ADC values can indicate higher tumour cellularity however with special precaution to various lesion components such as hematic, or lipid.^31^Chhabra et al concluded that diffusion-weighted imaging is helpful in tumour grading of soft tissue malignancies with good to excellent inter-reader reliability^7^

Higher maximum standardized uptake values in FDG PET CT are significantly associated with high-grade tumours; however, an overlap of these values can occur between low- and high-grade lesions.^32,33^ Additionally, FDG PET could significantly alter management of patients for staging and restaging.

In the study of Sagiyama et al, they performed multiparametric voxel-based analysis of standardised uptake values and apparent diffusion coefficients of soft tissue tumours with a positron emission tomography MR system and they concluded that it can be helpful to differentiate high-grade from low/intermediate-grade soft tissue masses.^34^

In our study, we did not use any automated texture analysis techniques; radiomics-based machine -earning models have shown promising results in the grading of soft tissue sarcomas;^35–38^ however, larger sample size is required with uniform imaging protocols for research, not yet available for clinical use. Li et al reported that a combination of several dynamic contrast-enhanced magnetic resonance imaging parameters can have a high diagnostic performance for differentiating between the different histological grades of soft tissue sarcomas (20). Lee et al analysis showed correlation between mean apparent diffusion coefficient value obtained from diffusion imaging with Ki-67 labelling index in soft tissue sarcoma, a marker indicating cellular proliferation.^39^

## Conclusion

A diagnostic score based on three signs, tumour heterogeneity on FS sequences, intratumoural and peritumoural enhancement, can be used to identify high-grade sarcoma. Patients who have two or three positive signs are at high risk of high-grade sarcoma and those with 0 or one positive sign are at low or moderate risk.
